# Childhood Adversity as a Predictor of Non-Adherence to Statin Therapy in Adulthood

**DOI:** 10.1371/journal.pone.0127638

**Published:** 2015-05-26

**Authors:** Maarit Jaana Korhonen, Jaana I. Halonen, M. Alan Brookhart, Ichiro Kawachi, Jaana Pentti, Hasse Karlsson, Mika Kivimäki, Jussi Vahtera

**Affiliations:** 1 Department of Pharmacology, Drug Development and Therapeutics, University of Turku, Turku, Finland; 2 Department of Public Health, University of Turku, Turku, Finland; 3 Finnish Institute of Occupational Health, Helsinki, Finland; 4 Department of Social and Environmental Health Research, London School of Hygiene and Tropical Medicine, London, United Kingdom; 5 Department of Epidemiology, University of North Carolina, Chapel Hill, North Carolina, United States of America; 6 Harvard School of Public Health, Boston, Massachusetts, United States of America; 7 Finnish Institute of Occupational Health, Turku, Finland; 8 Department of Clinical Science, University of Turku, Turku, Finland; 9 Department of Epidemiology and Public Health, University College of London, London, United Kingdom; 10 Turku University Hospital, Turku, Finland; Sookmyung Women's University, KOREA, REPUBLIC OF

## Abstract

**Purpose:**

To investigate whether adverse experiences in childhood predict non-adherence to statin therapy in adulthood.

**Methods:**

A cohort of 1378 women and 538 men who initiated statin therapy during 2008–2010 after responding to a survey on childhood adversities, was followed for non-adherence during the first treatment year. Log-binomial regression was used to estimate predictors of non-adherence, defined as the proportion of days covered by dispensed statin tablets <80%. In fully adjusted models including age, education, marital status, current smoking, heavy alcohol use, physical inactivity, obesity, presence of depression and cardiovascular comorbidity, the number of women ranged from 1172 to 1299 and that of men from 473 to 516, because of missing data on specific adversities and covariates.

**Results:**

Two in three respondents reported at least one of the following six adversities in the family: divorce/separation of the parents, long-term financial difficulties, severe conflicts, frequent fear, severe illness, or alcohol problem of a family member. 51% of women and 44% of men were non-adherent. In men, the number of childhood adversities predicted an increased risk of non-adherence (risk ratio [RR] per adversity 1.11, 95% confidence interval [CI] 1.01–1.21], P for linear trend 0.013). Compared with those reporting no adversities, men reporting 3–6 adversities had a 1.44-fold risk of non-adherence (95% CI 1.12–1.85). Experiencing severe conflicts in the family (RR 1.27, 95% CI 1.03–1.57]) and frequent fear of a family member (RR 1.27, 95% CI 1.00–1.62]) in particular, predicted an increased risk of non-adherence. In women, neither the number of adversities nor any specific type of adversity predicted non-adherence.

**Conclusions:**

Exposure to childhood adversity may predict non-adherence to preventive cardiovascular medication in men. Usefulness of information on childhood adversities in identification of adults at high risk of non-adherence deserves further research.

## Introduction

According to a recent meta-analysis [1], ~9% of cardiovascular events can be attributable to non-adherence to preventive cardiovascular medications. The estimated prevalence of non-adherence to statins, defined as taking <80% of the prescribed medication, was 46%, translating to 9 excess deaths due to cardiovascular disease per 100,000 Europeans offered statin therapy per year [1]. Multiple patient, physician, and health system-related factors as well as their interactions influence adherence to long-term medication [2, 3]. Suggested patient-related factors leading to non-adherence include presence of psychological problems, cognitive impairment, beliefs about the treatment and illness, and high cost of medication [2].

To date, several studies have shown that early-life socioeconomic adversities predict cardiovascular disease in adults [4, 5]. Furthermore, childhood adversity has been associated with the emergence of cardiovascular risk factors, such as high systolic blood pressure [6], obesity [7], type 2 diabetes [7], binge drinking [8], and smoking [9, 10], and higher overall cardiometabolic risk in adulthood [11]. Longitudinal studies suggest an increased risk of cardiovascular events among individuals reporting such childhood adversities as financial difficulties, interpersonal conflicts, long-term illness in the family [12], or physical abuse [13]. Moreover, childhood adversities are strongly linked with adulthood depression [14], another cardiovascular risk factor and a correlate of non-adherence [15, 16]. Accordingly, it seems plausible that the associations between childhood adversity and cardiovascular disease later in life may be partly mediated by medication non-adherence. To our knowledge, one previous study, including men only, has investigated the association between childhood adversity and non-adherence to cardiovascular medication in adulthood: low socioeconomic position in childhood, operationalized as father’s occupation at birth, was found to predict discontinuation of statin therapy in middle age [17].

In this study, we determined whether the number or type of childhood adversities predict non-adherence to statin therapy among new statin users in the Finnish Public Sector study cohort [18]. As there are sex-differences in cardiovascular disease rates [19] and adherence to cardiovascular medication [20], and the reasons for non-adherence may vary by sex [16, 21, 22], we stratified our analyses by sex.

## Materials and Methods

### Study population and design

**Fig 1:**
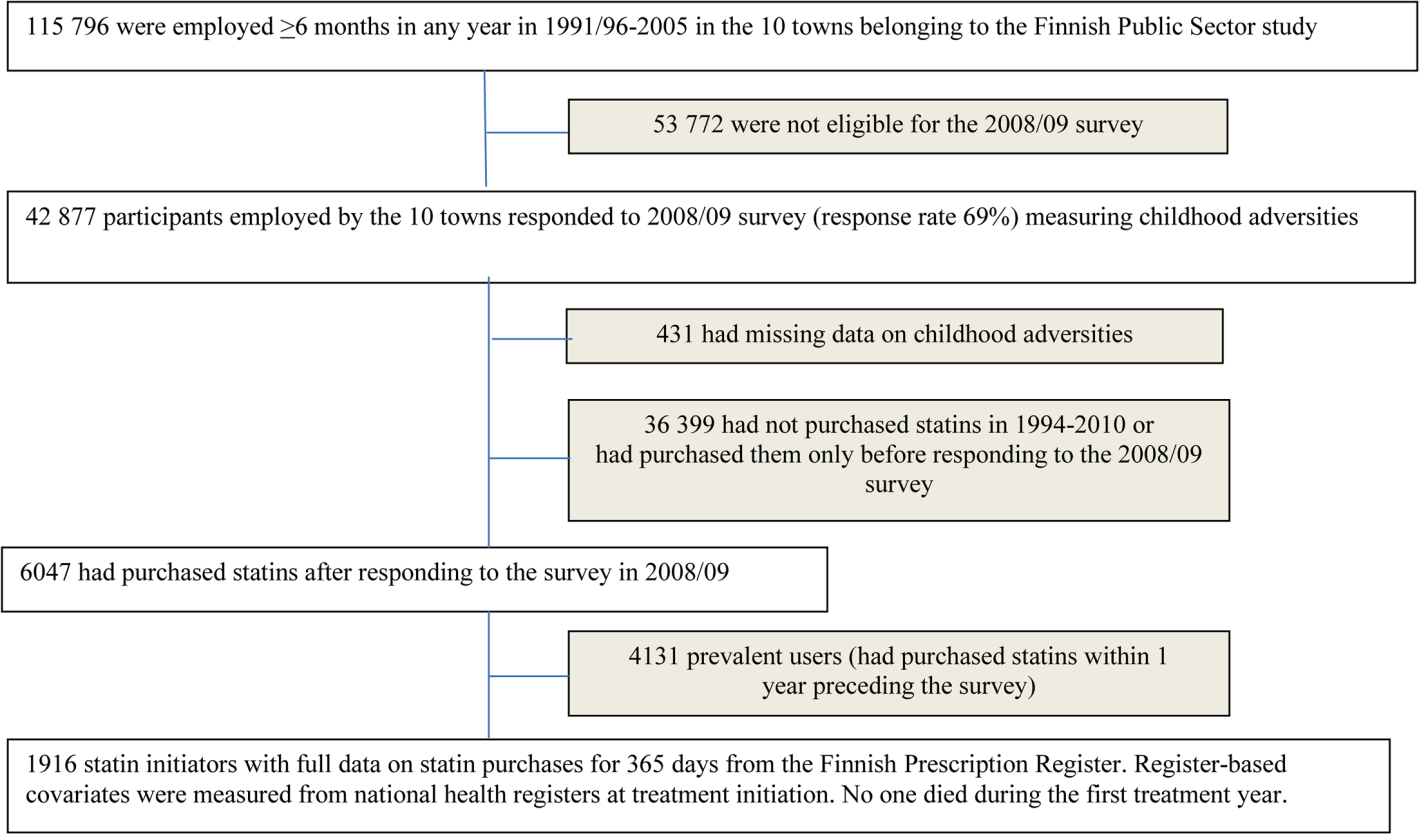
Chart of the sample selection.

The study’s register cohort includes all the employees of 10 towns and 6 hospital districts who had a ≥6-month job contract in 1991–2005. Nested survey cohorts, then, include all employees at the time of the surveys repeated by 4-year intervals since 2000. In 2008/2009, the self-administered questionnaire inquired information on childhood adversities; consequently, we first included those participants who were employed by the ten towns and responded to the survey in 2008/2009 (42,877 responded; response rate 69%). Using the unique personal identification numbers, we linked statin dispensation data [23] to the survey data and restricted the sample to those who initiated statin therapy after the survey. An initiator refers to an individual with no statin dispensations within one year preceding the survey. The final study sample included 1916 initiators with full data on statin purchases for a 12-month follow-up by December 31, 2011 (Fig 1).

### Assessment of childhood adversities

We assessed the occurrence of childhood adversities using six survey questions modified from Statistics Finland’s Survey of living conditions [24]. Respondents were asked whether they had experienced the following adversities: parents’ divorce/separation, long-term financial difficulties in the family, severe conflicts in the family, frequent fear of a family member, severe or chronic illness of a family member, and alcohol problem of a family member (response categories: no, yes, or cannot say). The items were analysed separately and as a summary variable (0, 1, 2, or 3–6 adversities). The category “cannot say” was coded as missing information when the items were analyzed separately. For the summary variable those with either type of missing information on an item were coded as not having that specific adversity. The reliability of this summary measure has been previously reported to be good (κ-values of responses with a 5-year interval from 0.56 to 0.90 [25]), and the measures has been shown to longitudinally predict, for instance, coronary heart disease [12] and depression [14].

### Assessment of adherence

The Finnish Prescription Register [23] is a pharmacy-claims database, managed by the Social Insurance Institution, provided data on statin use. The register contains records of all prescription drug purchases reimbursed to residents in non-institutional settings. For each drug, the dispensing date, the World Health Organization Anatomical Therapeutic Chemical (ATC) classification code [26], and the quantity dispensed are recorded. As the register contains information only on prescriptions that are both dispensed and reimbursed, we were not able to study primary non-adherence to statin therapy.

The outcome was non-adherence to statins (ATC code C10AA) during the 365 days after the initiation in 2008–2010. We measured adherence as the proportion of days covered by the dispensed tablets [27], on the assumption of a daily dose of 1 tablet [28]. We defined non-adherence as proportion of days covered <80% [1].

Finland has a universal drug reimbursement system [29]. All patients are entitled to a basic reimbursement which covered 42% of the price of statins during the study years. Patients with dyslipidaemia associated with coronary artery disease or familial hypercholesterolemia are an exception and, for them, 72% of the price of statins was reimbursed. Statin therapies were typically initiated with low-cost generic simvastatin (97% in 2008 [30]); thus, the impact of statin costs on non-adherence is likely to be small in our study.

### Covariates

We included sex, age, education, and marital status as sociodemographic covariates. Level of education, obtained from Statistics Finland, was classified as high (university or college degree) or low (vocational or basic education) [31]. Information on marital status (married or cohabiting vs. single, divorced, or widowed) and lifestyle-related risks came from the survey responses. Current smoking was defined as smoking (almost) daily. Heavy alcohol use was defined as consuming >210 grams of pure alcohol/week [32] based on the habitual frequency and amount of beer, wine, and spirits intake transformed into grams of pure alcohol. In addition, those respondents who reported that they had passed out due to heavy drinking during the past 12 months were classified as heavy alcohol users. Physical activity was measured by the Metabolic Equivalent Task index; the sum score of Metabolic Equivalent Task hours/day <2 indicated physical inactivity. Self-reported weight and height were used to determine body mass index and obesity (body mass index ≥30 kg/m^2^). A binary variable (any of the five risk factors vs. none) was created to indicate the presence of lifestyle-related risk.

Health indicators potentially affecting adherence [3] were extracted from the registers of the Social Insurance Institution and the Finnish Care Register [23]. Cardiovascular comorbidity was defined as entitlement to higher reimbursement for medication of chronic hypertension, heart failure, coronary heart disease, or diabetes at statin initiation, or as any hospitalization related to these conditions, stroke, or arrhythmia within 36 months prior to statin initiation. Depression was defined as hospitalization for depression or use of antidepressants (ATC code N06A) during 36 months before statin initiation.

### Statistical analyses

We stratified all analyses by sex. Statistical significance of the difference in the age distributions between sexes was tested with the Student’s t test and those in categorical variables with the Chi square test. We estimated the risk ratios (RR) for non-adherence and their 95% confidence intervals (CI) with log-binomial regression models. As the effect of childhood adversities on non-adherence may be partly mediated through adulthood socioeconomic status, comorbidities, lifestyle-related risks, and depression and the analyses adjusted for (or stratified by) these variables may be over-adjusted [33], the model was first adjusted for age at statin initiation only (Model 1) and then further for education and marital status (Model 2). Finally, the fully adjusted model (Model 3) included current smoking, heavy alcohol use, physical inactivity, obesity, depression and cardiovascular comorbidity in addition to the preceding variables (Model 3). Age was entered into the models as a continuous variable and all other covariates as categorical variables (dichotomized as described in the preceding section).

We first estimated the association between the four-class adversity measure (0, 1, 2, or 3–6 adversities) and non-adherence. The linear trend in this association was tested treating the number of adversities as a continuous variable. Second, we examined each type of adversity separately. The numbers of participants included in these analyses vary as those participants missing data on the adversity in question or covariates were excluded from the type specific analyses. Sex-differences were tested using the interaction term “sex*adversity”. To examine variations in the associations between the number of adversities and non-adherence across the subpopulations defined by age, education, marital status, presence of lifestyle-related risk, and comorbidity, we included the interaction term “subpopulation characteristic*number of adversities” in the age-adjusted models.

Two-sided *P* values <0.05 were interpreted as statistically significant. Statistical analyses were conducted with SAS 9.2 statistical software (SAS Institute, Inc., Cary, NC, USA).

### Ethics statement

According to the Finnish law, written consent is not required for register-based and survey research, as long as participation is voluntary. The participants of the Finnish public Sector study were informed about the aims of the study and the possible record linkages. Participants’ information was anonymized and de-identified prior to analysis. The Ethics Committee of the Helsinki and Uusimaa Hospital District approved the study.

## Results

We identified 1378 (72%) female and 538 (28%) male respondents who initiated statin therapy after the survey by December 31, 2010 (Table 1, S1 File). Among these initiators, 71% of women and 63% of men were free of cardiovascular comorbidities (*P* = 0.002). Compared to women, men were younger, more commonly married or cohabiting, current smokers and heavy alcohol users (*P* <0.001). Conversely, depression was twice as prevalent among women as among men (21% versus 10%, *P* <0.001).

**Table 1:** Baseline characteristics by sex.

Characteristic	Women (n = 1378)	Men (n = 538)	*P* value^a^
Age (year)^b^	56.8 ± 7.0 (29–75)	55.0 ± 8.0 (31–74)	<.0001
Education			0.86
Low	749 (54)	290 (54)	
High	629 (46)	248 (46)	
Married or cohabiting			<.0001
No	398 (29)	97 (18)	
Yes	970 (71)	435 (82)	
Current smoking			<.0001
No	1186 (87)	423 (79)	
Yes	183 (13)	111 (21)	
Heavy alcohol use^c^			<.0001
No	1248 (91)	369 (69)	
Yes	124 (9)	167 (31)	
Physical inactivity			1.00
No	858 (63)	336 (63)	
Yes	511 (37)	200 (37)	
Obesity			0.07
No	963 (73)	404 (77)	
Yes	364 (27)	123 (23)	
Depression^d^			<.0001
No	1091 (79)	486 (90)	
Yes	287 (21)	52 (10)	
Cardiovascular comorbidity^e^			0.002
No	972 (71)	340 (63)	
Yes	406 (29)	198 (37)	
Childhood adversities in the family			
Parents’ divorce	191 (14)	86 (16)	0.18
Financial difficulties	374 (30)	147 (30)	0.85
Severe conflicts	339 (27)	130 (26)	0.72
Fear of family member	255 (19)	74 (14)	0.012
Severe illness	494 (37)	167 (32)	0.07
Alcohol problem	339 (25)	127 (25)	0.77
Number of childhood adversities			0.66
0	494 (36)	204 (38)	
1	366 (27)	136 (25)	
2	203 (15)	85 (16)	
3–6	315 (23)	113 (21)	

Data are expressed as mean ± standard deviation (range) or number and proportion, n (%). The n do not sum up to 1378 for women and 538 for men for some variables because of missing data.

^a^Statistical significance of differences between women and men tested using the t-test for age and the Chi square test for categorical variables.

^b^ Age distributions presented also in Table A in S1 File.

^c^ >210 grams of pure alcohol/week and/or ≥1 extreme drinking occasions/past year

^d^Hospitalization for depression or use of antidepressants.

^e^Diabetes mellitus, hypertension, coronary insufficiency, coronary heart disease, cardiac arrhythmia, and/or cerebrovascular disease.

Two in three respondents reported one or more childhood adversities (Table 1). Severe illness of a family member was the most common adversity, 37% of women and 32% of men reporting it. The largest relative difference between the sexes appeared in reporting fear of a family member (19% in women and 14% in men, *P* = 0.012).

**Fig 2:**
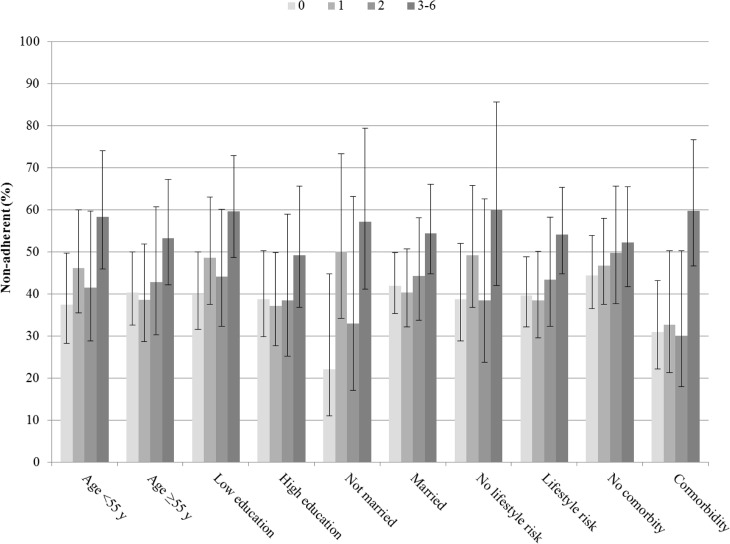
Risk of non-adherence (95% confidence interval) by the number of childhood adversities in subpopulations of men.

Overall, 237 (44%) of men and 700 (51%) of women were deemed non-adherent to statins during the first treatment year (*P* = 0.008). We found no association between the number of childhood adversities and the risk of non-adherence in women (Table 2). In men, however, the risk of non-adherence increased with the number of adversities (*P* for trend 0.013, *P* for interaction with sex 0.048). Specifically, those men with 3–6 adversities had a 1.44-fold risk of non-adherence (95% CI 1.12–1.85) compared with men reporting no adversities in the fully adjusted analyses (Table 2). As shown in Fig 2 (Table B in S1 File), the risk of non-adherence tended to increase with the number of adversities in all subpopulations of men with one exception. Among men with cardiovascular comorbidities, the risk of non-adherence remained low in men with 1–2 adversities; however, among men with 3–6 adversities, the risk of non-adherence almost doubled in comparison with those with no adversities (age-adjusted RR, 1.93, 95% CI 1.28–2.93). In contrast, among men without comorbidities, the corresponding age-adjusted RR was 1.18 (95% CI 0.88–1.58) (*P* for interaction 0.07).

**Table 2:** Risk ratios of non-adherence by the number of childhood adversities among statin initiators.

Number of adversities	Model 1	Model 2	Model 3
	RR (95% CI)	RR (95% CI)	RR (95% CI)
**Women** (n = 1299)			
0	1.00	1.00	1.00
1	0.99 (0.87–1.13)	0.99 (0.86–1.13)	1.02 (0.89–1.17)
2	0.93 (0.79–1.11)	0.94 (0.79–1.11)	0.95 (0.80–1.13)
3–6	1.05 (0.91–1.20)	1.05 (0.91–1.20)	1.05 (0.91–1.21)
linear trend	1.01 (0.96–1.06)	1.01 (0.96–1.05)	1.00 (0.96–1.05)
	*P* = 0.70	*P* = 0.71	*P* = 0.62
**Men** (n = 516)			
0	1.00	1.00	1.00
1	1.07 (0.82–1.39)	1.10 (0.85–1.43)	1.07 (0.82–1.40)
2	1.06 (0.79–1.44)	1.06 (0.79–1.44)	1.11 (0.81–1.50)
3–6	1.40 (1.10–1.78)	1.41 (1.11–1.79)	1.44 (1.12–1.85)
linear trend	1.11 (1.03–1.21)	1.11 (1.03–1.21)	1.11 (1.01–1.21)
	*P* = 0.010	*P* = 0.010	*P* = 0.013

Note: RR = risk ratio; CI = confidence interval

Non-adherence refers to proportion of days covered by statin therapy <80%.

Model 1: adjusted for age.

Model 2: adjusted for age, education, and marital status.

Model 3: adjusted for age, education, marital status, current smoking, heavy alcohol use, physical inactivity, obesity, depression, and cardiovascular comorbidity.

Contrasts derived from log-binomial regression analyses including the interaction term sex*the number of adversities. *P*-values for the interaction term 0.040 (Model 1), 0.038 (Model 2) and 0.048 (Model 3).

Age was entered into the models as a continuous variable and other covariates as dichotomous variables (see Table 1 for categories). Participants missing data on any covariate were excluded from the analyses.

Among women, none of the individual childhood adversities predicted the risk of non-adherence (Table 3). Among men, experiencing severe conflicts in the family and fear of a family member were associated with a significantly increased risk of non-adherence (fully adjusted RRs 1.27, *P <* 0.05, Table 3). The four other adversities were not significantly associated with the risk of non-adherence among men. Overall, adjustments for different sets of potential confounders and mediators had little impact on the observed associations (Tables 2 and 3).

**Table 3:** Risk ratios of non-adherence by the type of childhood adversity among statin initiators.

Type of adversity in the family	Model 1	Model 2	Model 3
	RR (95% CI)	RR (95% CI)	RR (95% CI)
**Women**			
Parents’ divorce (n = 1291)	0.98 (0.85–1.15)	0.98 (0.85–1.14)	0.99 (0.85–1.15)
Financial difficulties (n = 1172)	1.04 (0.92–1.18)	1.04 (0.93–1.18)	1.05 (0.92–1.18)
Severe conflicts (n = 1194)	1.10 (0.98–1.24)	1.10 (0.98–1.24)	1.10 (0.97–1.24)
Fear of family member (n = 1253)	1.07 (0.93–1.21)	1.07 (0.93–1.21)	1.06 (0.93–1.22)
Severe illness (n = 1269)	0.98 (0.88–1.10)	0.98 (0.88–1.10)	1.00 (0.90–1.12)
Alcohol problem (n = 1261)	1.06 (0.94–1.19)	1.06 (0.94–1.19)	1.05 (0.93–1.19)
**Men**			
Parents’ divorce (n = 505)	1.20 (0.94–1.51)	1.19 (0.94–1.51)	1.18 (0.93–1.49)
Financial difficulties (n = 473)	1.12 (0.91–1.38)	1.12 (0.91–1.38)	1.12 (0.91–1.39)
Severe conflicts (n = 480)	1.29 (1.05–1.59)	1.29 (1.05–1.58)	1.27 (1.03–1.57)
Fear of family member (n = 498)	1.30 (1.02–1.64)	1.30 (1.02–1.64)	1.27 (1.00–1.62)
Severe illness (n = 497)	1.17 (0.96–1.43)	1.17 (0.96–1.44)	1.17 (0.95–1.43)
Alcohol problem (n = 492)	1.15 (0.93–1.43)	1.15 (0.93–1.43)	1.15 (0.93–1.43)

Note: RR = risk ratio; CI = confidence interval

Non-adherence refers to proportion of days covered by statin therapy <80%.

Comparisons between those with a specific adversity versus no such adversity.

Model 1: adjusted for age.

Model 2: adjusted for age, education and marital status.

Model 3: adjusted for age, education, marital status, current smoking, heavy alcohol use, physical inactivity, obesity, depression, and cardiovascular comorbidity.

Age was entered into the models as a continuous variable and other covariates as dichotomous variables (see Table 1 for categories). Participants missing data on a specific type of adversity or any of the covariates were excluded from the analyses.

## Discussion

In this analysis of almost 2000 statin initiators in Finland, childhood adversity predicted non-adherence to statin therapy in men during the first treatment year. One in five men reported three or more childhood adversities and had a ~40% increased risk of non-adherence compared with men with no childhood adversities. Experience of conflicts in the family and fear of a family member, in particular, were associated with an increased risk of non-adherence. Women reported childhood adversities as frequently as did men; however, no associations were evident between the number of childhood adversities nor any specific adversity and non-adherence in women.

Our findings support previous observations on the high rates of non-adherence to statin therapy [1, 20] as well as on the sex-differences in the patterns of and reasons for non-adherence [16, 20, 21, 22, 34, 35]. In a cross-sectional survey of prescription drug users in Sweden, for example, men reported more commonly forgetting and changing the dosages than did women [22]. Women, conversely, reported more commonly that they filled prescriptions but did not take the medication. In a US survey [34], ~70% of lipid-lowering medication users reported some form of unintentional non-adherence during the previous six months, men reporting forgetfulness more commonly than women. Also the role of psychopathology in non-adherence may differ between men and women. One study found that the presence of anxiety or depression increased the likelihood of non-adherence to antihypertensive medication among older men but not among women [35]. We recently reported 9-year trajectories of adherence to antihypertensive medication before and after the onset of depression [16]. After the depression onset, men had a 1.5 times higher rate of “days-not-treated” compared to the years before depression while no difference was observed among women. In another study, we observed a 1.3-fold increase in the prevalence of poor adherence (proportion days covered <40%) to antihypertensive medication after retirement among men and women with hypertension; however, among patients with diabetes, the prevalence of poor adherence to antidiabetic medication increased only in men [21].

Although literature on the consequences of childhood adversities is abundant, few studies have investigated differences in the impact of childhood adversities on health outcomes and behaviours between sexes; the findings have been mixed with some [12, 14, 36, 37] but not all [6, 9, 10] showing sex-differences. Overall, women seem more susceptible to the effect of childhood adversities on cardiovascular disease [12], depression [14] multiple health problems [36] and premature death [37]; sex-differences in the associations between adversities and health behaviours have been less consistent [9, 10]. As pointed out by Fuller-Thompson et al. [10], further research is needed to clarify how childhood adversities influence health outcomes and health behaviours differentially by sex.

One potential explanation for the increased risk of non-adherence among those exposed to childhood adversities is that adherence to preventive medication, such as statin therapy, is an aspect of future orientation. In behavioural economics, the characteristic of undervaluing future benefit is referred to as present-focused preferences or myopia [38]. Present-focused orientation may emerge early in life because of adverse experiences; a child learns to focus on the present because the future is uncertain. Such orientation may persist into adulthood; myopia arises and leads to non-adherence because the patient is distracted by having to deal with more urgent needs, such as financial worries or balancing work-family responsibilities. Second, as intentional non-adherence may be regarded as risky behaviour [22], our observations are plausible in the light of the previous findings on the association between childhood adversity and engagement in risky behaviours, such as excessive alcohol consumption and promiscuity, in adulthood [39]. Third, unintentional non-adherence may result from impaired cognitive capacity [40], which has also been linked to early-life adversities [39]. Yet another possible route linking childhood adversity and non-adherence is attachment [41]. In patients with diabetes, dismissing attachment style has been associated with non-adherence to antidiabetic medications [42] and attachment styles characterized by low levels of collaboration have been associated with more missed primary care appointments compared to secure attachment style [43]. Finally, experiences of childhood maltreatment have been linked to high use of prescription medications [44] and emergency room services [36, 45]. None of these potential mechanisms, however, can explain why childhood adversities seem to have no association with non-adherence to statin therapy among women.

### Strengths and limitations

One previous study, including only men, has reported on the associations between childhood adversity and adherence to cardiovascular medication in adults [17]; we were able to expand the current knowledge to the associations in women and to those between a range of self-reported adversities and adherence. We used refill data in a closed pharmacy system to measure adherence instead of self-reported measures affected by recall and social desirability bias.

Nevertheless, we may have underestimated non-adherence if dispensed medications were not used, and we could not differentiate between intentional and unintentional non-adherence. We could not validate self-reporting of childhood adversities against any objective measures; however, previous research suggests that adversities are more likely to be under-reported than over-reported [46]. Furthermore we did not have information on severity of the adversities reported by the respondents nor on their experiences of more severe adversities such as neglect, physical or sexual abuse. Due to reliance on data from healthcare utilization databases, we may have underascertained comorbidities, and therefore our fully adjusted models may be confounded by untreated depression and cardiovascular diseases. Childhood adversities and non-adherence to cardiovascular medications are both known to affect survival [1, 37]; the associations observed among those who survived until the survey in 2008/09 and then initiated statin use may have partly resulted from the selection process. The most likely direction of the above biases is to attenuate the associations between childhood adversities and non-adherence. Due to the small sample size we cannot rule out meaningful associations among women. For example, the upper limit of the 95% CI of the RR for exposure to 3–6 adversities was 1.21. With the risk of non-adherence being ~50% among women, this RR would translate to ~10 percent unit increase in the risk of non-adherence. Our study population was a relatively homogenous sample of Finnish public-sector employees; the results could be different in other populations with different social and economic barriers to adherence.

Most importantly, we did not assess whether childhood adversity was associated with non-adherence over and above current adversity in the participants’ lives. Three types of causal models are postulated in life-course epidemiology: the latency model, the pathways model, and the accumulation model [47]. According to the latency model, early childhood adversity is hypothesized to directly affect the outcome (here, medication non-adherence in adulthood) regardless of adversities during later life (i.e., even if adverse circumstances stop). The reason is that present-focused preferences become entrenched in childhood and persist into adult life even if the adversity stops. According to the pathways model, early childhood adversity “begets” adult adversity; for example, family instability during childhood damages the child’s emotional development so that he is unable to form lifelong attachments later in life. In this instance, there is no direct effect of childhood adversity on the outcome; instead, it is completely mediated by adversity in adulthood. Finally, the accumulation model posits that both childhood and adult adversity affect the outcome, so that the effects are additive. Unfortunately, our study was not able to tease apart these alternative models.

## Conclusions

We found exposure to childhood adversities to predict an increased risk of non-adherence to statin therapy in men but not in women. Our findings imply that the effect of childhood adversities on cardiovascular disease in adulthood may be partly mediated by medication non-adherence. Further research is needed to confirm these findings in other populations, using different adversity measures and regarding adherence to other preventive medications and use of health services. In addition, further research is needed to determine the mechanisms underlying the association between childhood adversity and non-adherence in adults. Because childhood adversity may be a confounder in the association between non-adherence to cardiovascular medication and cardiovascular events and mortality [1], controlling for it could help reduce a “healthy adherer” bias [48] in future studies. Finally, since the available interventions on adherence are expensive and should be directed to patients at highest risk of non-adherence, information on childhood adversities could be useful in the development of a non-adherence risk score for identification of those patients.

## Supporting Information

S1 File Supporting information. Table A. Age distribution of men (n = 538) and women (n = 1378). Table B. Risk of non-adherence (95% confidence limits) in subpopulations of men (presented in Fig 2).
(DOC)
