# Supplementary material for: Childhood Adversity as a Predictor of Non-Adherence to Statin Therapy in Adulthood
Source: PLoS One. 2015 May 26;10(5):e0127638. doi: 10.1371/journal.pone.0127638 (PMC4444303; doi:10.1371/journal.pone.0127638)
Supplement: S1 File — Table A. Age distribution of men (n = 538) and women (n = 1378). Table B. Risk of non-adherence (95% confidence limits) in subpopulations of men (presented in Fig 2). (DOC) [file pone.0127638.s001.doc]

**S1 File. Supporting information**

**Table A. Age distribution of men (n=538) and women (n=1378)**

**Table B. Risk of non-adherence** (95% confidence limits) in subpopulations of men (presented in Fig 2)

|  |  |  |  |  |  |  |  |
| --- | --- | --- | --- | --- | --- | --- | --- |
|  | | | | | | | |

**Table A. Age distribution of men (n=538) and women (n=1378)**

Men Age (y) n %

31 1 0.19

32 1 0.19

33 1 0.19

35 1 0.19

36 2 0.37

37 5 0.93

38 4 0.74

39 3 0.56

40 5 0.93

41 8 1.49

42 6 1.12

43 12 2.23

44 9 1.67

45 8 1.49

46 19 3.53

47 13 2.42

48 18 3.35

49 26 4.83

50 19 3.53

51 17 3.16

52 28 5.20

53 19 3.53

54 13 2.42

55 20 3.72

56 19 3.53

57 25 4.65

58 29 5.39

59 23 4.28

60 31 5.76

61 35 6.51

62 26 4.83

63 19 3.53

64 16 2.97

65 15 2.79

66 12 2.23

67 7 1.30

68 5 0.93

69 7 1.30

70 5 0.93

71 3 0.56

72 1 0.19

73 1 0.19

74 1 0.19

Continued

**Table A. Continued**

Women Age (y) n %

29 1 0.07

30 2 0.15

31 1 0.07

32 1 0.07

33 1 0.07

35 2 0.15 *

36 2 0.15

37 2 0.15

38 6 0.44

39 3 0.22

40 9 0.65

41 5 0.36

42 10 0.73

43 13 0.94

44 17 1.23

45 14 1.02

46 13 0.94

47 30 2.18

48 26 1.89

49 36 2.61

50 40 2.90

51 49 3.56

52 56 4.06

53 70 5.08

54 63 4.57

55 77 5.59

56 72 5.22

57 78 5.66

58 81 5.88

59 82 5.95

60 78 5.66

61 80 5.81

62 78 5.66

63 64 4.64

64 54 3.92

65 34 2.47

66 35 2.54

67 32 2.32

68 14 1.02

69 22 1.60

70 11 0.80

71 6 0.44

72 5 0.36

74 2 0.15

75 1 0.07

| **Table B.** |  |  |  |  |  |  |  |  |  |  |  |
| --- | --- | --- | --- | --- | --- | --- | --- | --- | --- | --- | --- |
| **Risk of non-adherence (95% confidence limits) in subpopulations of men (presented in Fig 2)** | | | | | | | |  |  |  |  |
| Estimated by log-binomial regression. | | | |  |  |  |  |  |  |  |  |
|  |  |  |  |  |  |  |  |  |  |  |  |
|  | Risk of non-adherence (%) | | |  |  |  |  |  | |  |  |
| Number of Adversities | **Age**  **<55 y** | **Age**  **≥55 y** | **Low education** | **High education** | **Not married** | **Married** | **No**  **lifestyle risk** | **Lifestyle risk** | **No comorbity** | **Cormorbidity** | |
| 0 | 37.5011 | 40.32091 | 39.7444491 | 38.73990424 | 22.0976261 | 41.94965 | 38.74765299 | 39.58579 | 44.38804542 | 30.95301 |  |
| 1 | 46.15338 | 38.56982 | 48.61198892 | 37.10939552 | 50.0674044 | 40.35318 | 49.24314578 | 38.48891 | 46.65453734 | 32.67696 |  |
| 2 | 41.4617 | 42.85705 | 44.08282214 | 38.46582505 | 32.8834728 | 44.28607 | 38.51201174 | 43.37877 | 49.74799623 | 29.97519 |  |
| 3-6 | 58.33313 | 53.22723 | 59.5770353 | 49.14967263 | 57.1723383 | 54.39489 | 59.96554732 | 54.09113 | 52.27771527 | 59.83975 |  |
| Lower confidence limits (%) | | |  |  |  |  |  |  |  |  |  |
| 0 | 9.240452 | 7.771144 | 8.115583054 | 8.851511984 | 11.1822747 | 6.678333 | 9.90168614 | 7.46928 | 7.860370661 | 8.757938 |  |
| 1 | 10.65915 | 9.870596 | 11.12588935 | 9.452635427 | 15.903482 | 8.220608 | 12.40366247 | 8.92749 | 9.127180377 | 11.4118 |  |
| 2 | 12.64169 | 12.63203 | 11.79887268 | 13.35070176 | 15.7682176 | 10.49927 | 14.80975687 | 11.05606 | 12.02121627 | 12.10614 |  |
| 3-6 | 12.40535 | 11.07995 | 10.877466 | 12.38011789 | 16.0245497 | 9.605547 | 18.00330649 | 9.328653 | 10.52893708 | 13.1292 |  |
| Upper confidence limits (%) | | |  |  |  |  |  |  |  |  |  |
| 0 | 12.26183 | 9.63 | 10.20293763 | 11.47 | 22.6379999 | 7.942821 | 13.30575072 | 9.201519 | 9.55183959 | 12.21804 |  |
| 1 | 13.86016 | 13.2706 | 14.43436208 | 12.68837359 | 23.3066349 | 10.31865 | 16.57332916 | 11.62357 | 11.34703886 | 17.54087 |  |
| 2 | 18.18687 | 17.91137 | 16.11102776 | 20.44767021 | 30.2954146 | 13.76193 | 24.05700316 | 14.83781 | 15.84508268 | 20.30294 |  |
| 3-6 | 15.7561 | 13.986 | 13.29975317 | 16.54843921 | 22.2651328 | 11.66556 | 25.72737076 | 11.27278 | 13.17776271 | 16.81183 |  |
|  |  |  |  |  |  |  |  |  |  |  |  |
